# Determinants of health-related quality of life among individuals with opioid use disorder, recently released from incarceration

**DOI:** 10.1186/s13722-023-00375-0

**Published:** 2023-05-25

**Authors:** Techna Cadet, Ali Jalali, Philip J. Jeng, Sabrina Poole, George Woody, Sean M. Murphy

**Affiliations:** 1 Department of Population Health Sciences, Weill Cornell Medical College, 425 East 61St Street, Suite 301, New York, NY 10065 USA; 2 Perelman School of Medicine, University of Pennsylvania, Philadelphia, PA USA

## Abstract

**Background\Objectives:**

Concomitant with low rates of pharmacotherapy for incarcerated individuals with OUD, there is a high rate of opioid overdose following re-entry into the community. Our research objective was to develop a better understanding of the factors that influence health-related quality-of-life (HRQoL) among this population during the high-risk transition period from incarceration to community. Few studies have assessed health-related quality-of-life (HRQoL) among individuals with OUD who are involved with the criminal-legal system, let alone over the period directly surrounding release from incarceration.

**Methods:**

Secondary longitudinal analysis of data from a clinical trial where participants were randomized 1:1 to pre-release extended-release naltrexone (XR-NTX) + referral to community XR-NTX, vs. referral only. We conducted individual, multivariable regressions of EQ-5D domains (mobility, pain/discomfort, anxiety/depression; usual activities and self-care were excluded due to insufficient variation in scores), and the overall preference/utility score. HRQoL data were subset to timepoints immediately before release (baseline) and 12 weeks post-release; treatment groups were collapsed across condition. Multiple imputation by chained equations was conducted to handle missing 3-month data in the dependent variables and covariates, ad hoc.

**Results:**

Greater severity in the psychiatric composite score was associated with substantially lower HRQoL, across all measures, following release from incarceration. Greater severity in the medical composite score was associated with lower pain/discomfort-related HRQoL.

**Conclusions:**

Our findings highlight the importance of ensuring individuals with OUD are linked not only to MOUD, but also treatment for their comorbid conditions upon release from incarceration.

**Supplementary Information:**

The online version contains supplementary material available at 10.1186/s13722-023-00375-0.

## Introduction

The U.S. Department of Justice estimated the number of incarcerated individuals under state and federal jurisdiction to be 1,430,800 at the end of 2019 [1], approximately 15% of whom were believed to have an opioid use disorder (OUD) [2, 3]. Concomitant with low rates of medication treatment for incarcerated individuals with OUD (only ~ 5% receive buprenorphine, methadone, or naltrexone) [2, 3, 5], there is a high rate of opioid overdose following re-entry into the community. According to Binswanger et al., the risk of death within the first 2 weeks of re-entry into the community is 12.7 times greater for those with an OUD, than the general U.S. population [6].

Initiation on OUD pharmacotherapy just prior-to, or immediately-following release from incarceration has been associated with increased rates of entry to community-based treatment [6, 8, 9, 10, 11], treatment retention [9, 11, 13], and opioid abstinence [8, 10, 13, 14]. Evidence-based treatment for OUD has also been associated with reductions in violent behavior and criminal-legal activity [13, 16, 17, 18, 19, 20, 21, 22]; healthcare cost-offsets from fewer emergency department visits and inpatient admissions; [15, 17, 21, 22] improved workplace productivity and higher health-related quality-of-life (HRQoL) [19, 22, 24]. Although, not as much existing evidence speaks to extended-release naltrexone (XR-NTX) specifically, prior studies have shown it to be effective at reducing opioid use in real-world settings [21, 22, 25], including among persons with criminal-legal history [26]. Of particular relevance is Lee et al. [26], a randomized controlled-effectiveness trial comparing XR-NTX to treatment-as-usual (brief counseling and referrals to community treatment programs) among individuals with criminal-legal history, but not necessarily incarceration. Results of the study found that individuals who received XR-NTX had a lower rate of opioid relapse compared to the treatment as usual group.

HRQoL is typically measured with self-report instruments designed to capture an individual’s well-being across multiple domains (e.g., physical, mental, emotional, etc.). Some HRQoL instruments are capable of generating an index value, based on the respondent’s combination of domain scores, which is intended to reflect their specific health-state at that point in time [27]. Those index values can then be mapped to weights reflecting the general population’s preference/perceived-utility for that health-state. The HRQoL preference/utility weights generally vary between 0, signifying death, and 1, signifying optimal health; although, some instruments, such as the EuroQoL-5D (EQ-5D), are capable of measuring health-states perceived to be worse than death, thus resulting in a negative utility weight. The utility weight can also be used to calculate quality-adjusted life-years (QALYs) gained. The QALY is a longitudinal combination of an individual’s HRQoL for a particular health-state and the amount of time spent in that health-state; thus, it effectively provides a summary HRQoL score over a given time frame, using multiple data points, and is interpreted in the context of 1 QALY represents one year of perfect health [28]. The EQ-5D also includes a visual analog scale where respondents rate their overall health on a 0–100 scale, where 0 represents the worst health the person could imagine, and 100 the best [29].

Few studies have assessed HRQoL or QALYs among individuals with OUD who are also involved with the criminal-legal system, let alone over the time period directly before and after release from incarceration. Togas and colleagues examined the relationship between HRQoL, measured via the EQ-5D, and a variety of sociodemographic, health-related, and prison/facility characteristics, among the general male prison population in Greece [30]. On average, respondents indicated having some/extreme issues with anxiety/depression, but little-to-no issues in relation to the mobility, self-care, usual activity, and pain/discomfort [30]. Murphy et al. tested QALYs gained as part of a cost-effectiveness analysis conducted alongside a clinical trial comparing extended-release naltrexone (XR-NTX) to detoxification and referral to treatment, among community-dwelling individuals with OUD, who were also involved with the criminal-legal system, but had not necessarily been incarcerated [31]. The findings indicated that QALYs gained by participants in the XR-NTX arm surpassed those in the detox/referral arm at 25 and 78 weeks, with statistical significance at 25-weeks [31].

A recent trial by Woody et al. [32] studied outcomes associated with administering XR-NTX to detoxified prisoners with OUD before release, with referral to a nearby program for continuing XR-NTX after release, vs. referral to the same program to start XR-NTX after release with continued XR-NTX for 3 or more months. The primary outcome was relapse to opioid use at post-release month 3. HRQoL data were collected using the EQ-5D at baseline (pre-release), and at 12- and 24-weeks post-release. The pre-release XR-NTX arm self-reported a significantly-lower, mean 12-week score on the HRQoL visual analog scale (unadjusted overall health) compared to the XR-NTX referral arm [32]; however, the cost-effectiveness analysis conducted alongside the study found that the EQ-5D generated QALYs did not differ significantly between the two arms at 12- or 24-weeks, after controlling for potential confounding variables [33]. The individual EQ-5D domains (mobility, self-care, usual activities, pain/discomfort, anxiety/depression) were not tested for between-arm differences.

Our research objective was to develop a better understanding of the factors that influence both the individual components, and the overall HRQoL among individuals with OUD during the high-risk transition period from incarceration to community. These nuanced findings could identify characteristics that would not only inform policies to reduce the threat of opioid overdose among this population, but also improve their general well-being during this critical period.

## Methods

### Study design and data

We conducted a secondary analysis of HRQoL among persons with OUD who were transitioning from incarceration to the community. Our analysis focused on the determinants of specific EQ-5D HRQoL domains, as well as the overall preference/utility score (please see description in Introduction), over the first 12 weeks post-release, with the first measure taken just before release, and treatment groups collapsed across condition.

Data were obtained from the aforementioned Woody, et al. study, a 1:1 randomized trial, where participants with OUD who were incarcerated in the Philadelphia Department of Prisons were selected to receive either XR-NTX before release, with referral for continuing XR-NTX at a nearby community program after release (n = 74), or referral to start XR-NTX post-release, and receive continuing care at the same nearby program (n = 72) [32]. The Philadelphia Department of Prisons is actually the county jail, and at the time of the study was the sixth largest correctional facility in the U.S., consisting of 6 facilities with 800–1,200 incarcerated individuals each. Moreover, both settings were only approximately 2 miles from the Kensington area of Philadelphia, which has received much national attention for its prevalence of OUD and the related adverse consequences, such as overdose deaths and people experiencing homelessness [34, 35].

Participants for the study had to meet the following inclusion criteria: be 18 years of age, or older; have a DSM-V diagnosis of OUD; be interested in and not allergic to XR-NTX treatment; plan to reside in Philadelphia for the next 6 months; be eligible for Medicaid or other third-party health insurance; anticipate release from incarceration within 3–4 months, with no subsequent sentence longer than 30 days scheduled at a different carceral facility; no residential treatment stay for over 30 days after release; give consent to be contacted; be proficient in English (speaking and reading); provide contact information of 3 references that could help locate the participant; not pregnant or breastfeeding; be eligible for treatment at the community treatment provider (NET Steps); be able to answer 9 out of 10 items on a study quiz,with three attempts allowed; no neurological, cardiovascular, renal, or hepatic conditions that could make participation hazardous; no active tuberculosis; not experiencing psychosis, suicidal thoughts, or homicidal tendencies; no uncontrolled seizure disorder; not on opioid therapy for chronic pain; and were not already sentenced to naltrexone.

Data were collected from participants at baseline (pre-release), and at 12- and 24-weeks post-release. However, of the 146 randomized participants, 60 were lost after randomization due to unforeseen facility transfer, withdrawing consent, release prior to scheduled study induction, or becoming otherwise ineligible for study participation, leaving a final analytic sample of 86 (n = 38 pre-release XR-NTX; n = 48 referral only) [32]. Moreover, uncertainty surrounding release dates led to 12 pre-release participants requiring a second, “reset” dose before community reentry. Baseline characteristic comparisons between the 146 randomized participants and the resulting 86 subset of participants is described in Woody et al. who noted that both samples achieved mean balance. Further comparisons among participants lost after randomization could not be made. Additional details on the parent study’s design and findings can be found in Woody et al. [32].

The parent study measured HRQoL via the EuroQol 5D (EQ-5D-5L) [29, 36, 37]. The EQ-5D measures current HRQoL across 5 dimensions (mobility, self-care, usual activities, pain/discomfort, anxiety/depression), and is one of the most widely-used generic, preference-based HRQoL instrument [38]. The EQ-5D-5L has 5 possible categorical responses (or levels) for each domain, ranging from the best possible health (e.g., “I have no problems walking”) to the worst possible health (e.g., “I am unable to walk”) [29]. As discussed in the Introduction, the EQ-5D-5L preference/utility score represents the general-public’s perceived quality of a given health state, defined via combinations of the individual domain scores. The EQ-5D-5L HRQoL preference/utility score ranges from − 0.573 to 1, where 0 is a state considered comparable to death, 1 implies perfect health, and a negative value implies a state perceived to be worse than death [39].

Our outcome variables were the following longitudinal measures: a) categorical HRQoL measures of mobility, pain/discomfort, and anxiety/depression (usual activities and self-care were excluded because 97% and 98% of participant responses indicated “minimal difficulties” in these areas, respectively); and b) the previously-described preference/utility score associated with the participant’s health state. For ease of interpretation, the EQ-5D domain values were recoded such that an increase in values aligned with an improvement in the relevant health condition, similar to the utility score. As mentioned above, our analysis focused solely on the first two data collection timepoints, baseline and 12 weeks post-release, as the purpose of this study was to conduct an in-depth evaluation of the factors that influence HRQoL over this critical transition period for persons with OUD.

Regression covariates included baseline sociodemographic variables (age, race, gender, high school completion, health insurance status), lifetime and past 30 days incarceration, randomization arm, and time-varying composite scores from the Addiction Severity Index (ASI) Lite (medical, employment/support, alcohol, drug, legal status, family/social relationships, psychiatric) [40]. The ASI Lite is an instrument designed to identify problem areas in individuals with substance use disorder (SUD), via semi-structured interviews inquiring about the 30 days prior to assessment. The ASI composite score variables were continuous and ranged from 0 to 1. An increase in the ASI composite score indicates increased severity in the associated construct and have been shown to be associated with HRQoL in prior studies [38–42]. The time-varying ASI composite scores were advantageous to the research objective of evaluating determinants of HRQoL surrounding release from incarceration, given the theoretical importance of the information contained therein, and their proximity to the outcomes of interest.

Additional predictors included injection drug use, and time free from opioids (measured in weeks). History of injection drug use was included in the analysis since prior studies have found significant associations between it and depressive components of various HRQoL scales, as well as between HRQoL and other adverse effects of injection drug use (e.g. abscess) [43, 44]. Time free from opioids was added as a covariate to the analytical model, as prior studies have shown effective treatment for OUD resulting in time free from opioids, to be associated with improved HRQoL [21, 22, 45]. As in Jalali et al. [33], the weeks free from opioids variable was calculated as a combination of urinalysis and self-report of opioid use; missing urinalysis (measured weekly) or urinalysis that was refused were counted as positive with the exception where XR-NTX was administered within the past 28 days [46]. As a result of missingness in both our predictors and outcome variables, multiple imputations were conducted on the 4 EQ-5D-5L outcome variables (mobility, pain/discomfort, and anxiety/depression, preference/utility score) and 7 (ASI) Lite variables (medical, employment/support, alcohol, drug, legal status, family/social relationships, psychiatric).

### Analysis

Separate, multivariable regression models were run for the three HRQoL domains with sufficient variation (mobility, pain/discomfort, anxiety/depression), and the overall utility score, between baseline (pre-release) and 12 weeks post-release; all covariates mentioned above were included in each regression model. Ordinal logistic regression was chosen to assess the HRQoL domains, given their ordered categorical nature [47, 48]. Overall preference/utility was analyzed using a linear model after evaluating the most appropriate family and link functions within a generalized linear model framework, according to current guidelines, which recommend the assistance of the modified Parks test for family, and the Pregibon-link, Pearson-correlation, and modified-Hosmer-and-Lemeshow tests for the link function [49]. All models clustered standard errors at the participant level to account for within-subject correlation, and all analyses were conducted using Stata 17.0.

### Addressing missing data

The study was subject to significant patient attrition. Prior analyses of the parent study data concluded that data was missing at random (MAR), and employed robust methods such as multiple imputation, to avoid bias [32, 33, 50, 51]. The current study, which measured participant outcomes at baseline and 12 weeks, has zero missing data at baseline and 64% of missing data at the 12 week follow-up; additional details are available in the Supplementary Statistical Appendix. We conducted a battery of statistical tests that suggested the data were again MAR (see Additional file 1). Multiple imputation has been shown to perform well in addressing missing data bias when data are MAR [47, 53, 54, 55], and was recently suggested as an efficient approach of addressing missingness in trial-based studies evaluating costs and effectiveness measures, including QALYs [56]. Given the missingness in variables of interest on both sides of our empirical model, the multiple imputation by chained equations (MICE) approach was used to generate the final analytic dataset for each regression. MICE is a commonly-used, robust missing data technique which involves imputing missing information via sequential regressions where observed values for each variable with missingness are regressed on other variables in the model, and missing observations are replaced with predicted values from the regression [52, 53, 57]. One cycle is considered complete once the process has been repeated for each variable with missingness. This process is repeated for a pre-defined number of cycles, resulting in that number of “complete” datasets, the results of which are then pooled to create a final analytic dataset [52, 54]. Further detail on the missing data mechanism, imputation procedure, and model validation is provided in the Supplemental Statistical Appendix.

## Results

### Sociodemographic characteristics

Summary statistics of baseline participant characteristics are displayed in Table 1. Participants were 62% non-Hispanic White/Caucasian, 20% non-Hispanic Black/African American, and 16% Hispanic (Puerto Rican). On average, the participant population was 38 years old, with 79% having completed high school, 78% having health insurance, 13% being married, and 63% having a history of injection drug use. The average number of months spent incarcerated over the participant’s life was 53 (with 24% of responses censored at 99), including 27 days in the prior 30.

**Table 1:** Baseline Participant Characteristics

Sociodemographic Characteristic	Value (n = 86)
Race, %	
Non-Hispanic White/Caucasian	62%
Non-Hispanic Black/African American	20%
Hispanic-Puerto Rican	16%
Male	73%
Age, Mean (SD)	38 (8.65)
High School Completion, %	79%
Injection Drug Use	63%
Health Insurance	78%
Married	13%
Lifetime Incarceration^d^, Mean (SD)	53 (38.5)
Past 30 Days Incarceration, Mean (SD)	27 (8.45)
Weeks Abstinent, Mean (SD)	3 (0.11)
EQ-5D Characteristics, Mean (SD)	(Min, Max)
Mobility^a^	3.79 (0.62) (1, 4)
Anxiety/Depression^a^	2.74 (1.16) (0, 4)
Pain/Discomfort^a^	3.27 (1.03) (0, 4)
HRQoL Utility Score^b^	0.84 (0.20) (-0.073, 1)
ASI Characteristics, Mean (SD)	(Min, Max)
Medical^c^	0.14 (0.29) (0, 1)
Employment/support^c^	0.80 (0.21) (0.25, 1)
Alcohol^c^	0.10 (0.20) (0, 0.90)
Drug^c^	0.41 (0.11) (0.13, 0.72)
Legal^c^	0.45 (0.25) (0, 0.88)
Family/Social Support^c^	0.26 (0.28) (0, 0.9)
Psychiatric^c^	0.34 (0.23) (0, 0.73)

^a^The EQ-5D domain scores (mobility, anxiety/depression, pain/discomfort) range from 1–5, with 1 being the worst possible relevant status (e.g., “I am unable to walk”) and 5 being the best (e.g., “I have no problems walking”)

^b^The HRQoL utility score ranges from -0.573 to 1, where 0 is a health-state considered comparable to death, 1 implies perfect health, and a negative value implies a state perceived to be worse than death

^c^The ASI composite score variables ranged from 0 to 1, with 0 indicating no issues and 1 severe issues in the associated construct

^d^The lifetime incarceration variable is represented in months

### Significance between HRQOL domains and predictor variables

Table 2 displays the results of the multivariable regressions. The ASI psychiatric composite score was negatively associated with the mobility (OR = 0.68; p = 0.05) and anxiety/depression (OR = 0.52; p < 0.001) domains of the EQ-5D. As prior noted, ASI composite scores range from 0–1, 1 being most severe; thus, for ease of interpretation, we converted the odds ratios (ORs) to align with a 0.1 unit increase in the composite score. Therefore, a 0.1 unit increase in the psychiatric composite score was associated with a 32% decrease in the odds of improved mobility, and an 48% decrease in the odds of improved anxiety/depression.

**Table 2:** Multivariable Regression Results

Characteristics	Mobility			Anxiety/Depression			Pain/Discomfort			HRQOL Utility Score		
	Odds Ratio	SE	P-value	Odds Ratio	SE	P-value	Odds Ratio	SE	P-value	Coefficient	SE	P-value
ASI Components												
Medical	0.86	0.11	0.21	0.98	0.07	0.74	0.77	0.07	**0.001**	− 0.01	0.01	0.06
Employment/Support	0.88	0.15	0.45	0.93	0.08	0.41	1.04	0.11	0.73	0.00	0.01	0.77
Alcohol	0.77	0.15	0.19	0.86	0.11	0.23	1.07	0.17	0.68	− 0.01	0.01	0.25
Drug	0.63	0.27	0.29	0.99	0.22	0.96	1.01	0.24	0.98	− 0.01	0.02	0.47
Legal	1.24	0.21	0.21	0.93	0.08	0.38	1.02	0.10	0.86	0.00	0.01	0.75
Family/Social Relationship	1.11	0.15	0.42	1.11	0.10	0.23	1.15	0.11	0.16	0.02	0.01	**0.01**
Psychiatric	0.68	0.13	**0.05**	0.52	0.06	**0.001**	0.69	0.07	**0.001**	− 0.04	0.01	**0.001**
Sociodemographic												
Age	0.97	0.06	0.63	1.00	0.03	0.96	0.96	0.03	0.21	0.00	0.00	0.35
High School Completion												
Completed High School	0.20	0.23	0.17	0.84	0.42	0.72	0.93	0.57	0.90	− 0.03	0.04	0.41
Health Insurance												
Has Health Insurance	0.78	0.90	0.83	0.42	0.23	0.11	0.24	0.16	**0.04**	− 0.07	0.04	0.10
Race												
White	1.20	1.21	0.85	0.65	0.32	0.39	0.49	0.28	0.21	-0.04	0.04	0.27
Gender												
Female	1.07	1.22	0.95	1.16	0.57	0.77	0.65	0.40	0.49	0.00	0.04	0.97
Has IDU History	1.66	1.57	0.60	1.93	1.01	0.21	3.09	1.80	0.053	0.07	0.04	0.10
Lifetime Incarceration	0.99	0.01	0.55	1.01	0.01	0.44	0.99	0.01	0.12	0.00	0.00	0.99
Past 30 days incarceration (BL)	1.03	0.06	0.60	1.00	0.02	0.96	0.99	0.04	0.77	0.00	0.00	0.75
Randomization group												
Referral Only	0.10	0.11	**0.03**	1.90	0.89	0.17	0.76	0.42	0.62	− 0.02	0.04	0.61
12-week Visit	0.10	0.16	0.14	0.76	0.62	0.73	1.46	1.39	0.69	− 0.08	0.07	0.26
Weeks Abstinent	0.77	0.31	0.51	1.01	0.26	0.96	1.04	0.32	0.89	0.02	0.02	0.32

The first 3 HRQoL Domains were analyzed using ordinal logistic regression and the HRQoL utility score was analyzed using OLS. IDU denoted injection drug use. Bolded values denotes statistical significance.

Pain/discomfort was significantly associated with ASI medical (OR = 0.77; p < 0.001), ASI psychiatric (OR = 0.69; p < 0.001), and health insurance (OR = 0.24; p = 0.04). Specifically, a 1/10^th^ of a unit increase in the ASI medical and psychiatric composite score was associated with a 23% and 31% decrease in the odds of improved pain/discomfort, respectively. Having health insurance was associated with a 76% decrease in the odds of experiencing improved pain/discomfort (p = 0.04). The relationship between health insurance and pain/discomfort was then further explored by regressing several potentially-explanatory variables (prescribed medication regularly; bothered by medical problems; hospital admission; medical office visit; and days experienced medical problems) on health insurance via both logistic regression and ordinary least squares (OLS), with standard errors clustered at the participant level. Logistic regression was conducted for the categorical variables (prescribed medication regularly; bothered by medical problems; hospital admission; medical office visit) and OLS was conducted on the continuous variable (days experienced medical problems). Results (Appendix Table 4) showed that health insurance was significantly associated with regular medication prescription (OR = 0.49; p = 0.01) hospital admission (OR = 0.10; p < 0.001), medical office visit (OR = 0.29; p < 0.001), and days experienced medical problems (Coefficient = 4.72; p < 0.001).

The ASI psychiatric composite score was inversely related to overall utility (p < 0.001), as measured via the EQ-5D, with a 1/10^th^ of a unit increase in the ASI psychiatric score being associated with a 0.04 unit decrease in utility. Conversely, a 1/10th of a unit increase in the ASI family/social relationship composite score was associated with a 0.02 unit increase in utility (p = 0.01).

## Discussion

Increased psychiatric complications, as measured by the ASI, were strongly associated with reduced HRQoL scores over the period immediately following incarceration, in all domains explored (mobility, anxiety/depression, pain/discomfort), as well as a relatively large decrease in overall utility. Although the ASI does not diagnose psychiatric illness, it does target problematic mental health areas in individuals with substance use disorder. Other studies have found a similar association between HRQoL and mental health among individuals with SUD, including OUD, [38, 59, 60, 61] as well as associations between mental health disorders and pain among individuals with OUD [62, 63]. These results are indicative not only of the debilitating effects of untreated/undertreated mental health disorders, which are highly prevalent among both persons with OUD and those engaged in the criminal-legal system, but also the need for linkage to community mental health services immediately following release from incarceration. In a 2016 study by Begun et al., approximately 63% and 57% of incarcerated individuals anticipated needing some sort of substance use and mental health treatment during community reentry, respectively, but only 21% and 15% reported receiving professional help for such services within 4 months post-release [64]. According to the sequential intercept model (SIM), which describes how individuals with mental health and substance use disorders move through the criminal-legal system, steps such as pre-release planning, warm hand-offs (i.e., individuals who are transported directly to treatment services upon release), peer support services, etc., can increase engagement in mental health services, and improve psychiatric health [65], which our results suggest may in-turn improve quality-of-life.

Increased severity of one’s medical condition was associated with a significant reduction in their HRQoL within the pain/discomfort domain, and was negatively correlated with their overall utility, but just outside of the traditional 5% threshold at p = 0.06. Given the high prevalence of chronic conditions among persons with OUD, particularly among those who are incarcerated, the results are not necessarily surprising. This is representative of previous literature which has shown that OUD is often associated with comorbid medical conditions, including chronic pain [66, 67]. Previous literature has also showed chronic conditions are not managed well in jail/prison settings [68]. Additionally, state and federal prisoners have significantly higher prevalence rates of ever having chronic diseases compared to the general population: hypertension (30.2% vs. 18.1%); diabetes/high blood sugar (9% vs. 6.5%); heart-related problems (9.8% vs. 2.9%); stroke-related problems (1.8% vs. 0.7%); and asthma (14.9% vs. 10.2%) [69]. Infectious diseases are also more prevalent among incarcerated individuals than the general population, with incarcerated Individuals 3 times more likely to have HIV/AIDS [70].

Additionally, individuals with health insurance were more likely to have worse HRQoL in the pain/discomfort domain. As this result was not expected, further bivariate analyses were conducted to explore the potential causal mechanisms (Appendix Table 4). We found that although participants with and without health insurance did not differ significantly with regard to how much their medical problems bothered them, those with health insurance experienced 4.5 more days of medical problems out of the past 30, on average (p < 0.001); however, those with health insurance were less likely to have been prescribed medication on a regular basis (p = 0.01), visited a medical office recently (p < 0.001), or been admitted to the hospital recently (p < 0.001). The results of the initial analysis could potentially be explained by the concept of adverse selection in health economics, in which individuals who are sicker or pose higher risk to the insurer are more likely to have health insurance, whereas healthier individuals may not have health insurance because they don’t necessarily feel like they need it [71]. This may be particularly true of individuals leaving incarceration who have the opportunity to immediately (re)enroll in Medicaid, regardless of pre-existing conditions [72].

Although not significantly associated with any of the individual HRQoL domains, family/social relationship issues were associated with slightly higher utility. This finding is in contrast to previous studies examining family/social relationships and HRQoL, which indicated that as family/social relationships improve, HRQoL improves as well, including among individuals undergoing treatment for OUD/SUD [59, 73].

The primary strengths of this study were the longitudinal and detailed nature of the data, which allowed us to analyze determinants of HRQoL at different time points surrounding an individual’s release from incarceration. Limitations of this study included the small sample size (n = 86), and the extent of missing data for the 12-week follow-up period. As discussed in the methods section and supplementary appendix, MICE was used to minimize potential bias arising from the missing data, and sensitivity analyses were conducted to evaluate alternative imputation methods. Furthermore, the racial/ethnic make-up of the study population was 62% Non-Hispanic White/Caucasian, 20% Non-Hispanic Black/African American, and 16% Hispanic-Puerto Rican, whereas according to the August 2022 Philadelphia Prison Population Report, the racial/ethnic make-up was 72.4% Black, 17% Latinx, and 9.3% White. This discrepancy likely limits the generalizability of our findings both within and outside of the Philadelphia Department of Prisons; however, there is little-to-no information regarding the racial/ethnic makeup of the incarcerated population with OUD.

## Conclusion

Our findings highlight the importance of ensuring individuals with OUD are linked not only to MOUD, but also treatment for their comorbid conditions, particularly mental health disorders, upon release from incarceration. A holistic treatment approach for these individuals may be especially beneficial, given the estimated additive, independent effects of medical and psychiatric conditions on overall utility, as well as the potential benefits associated with care coordination [72]. To our knowledge, this study is the first of its kind to conduct an in-depth HRQoL analysis, exploring individual HRQoL domains over the period immediately surrounding release from incarceration among persons with OUD, while controlling for post-release treatment success, as well as other important factors associated with recovery from addiction and reentry into the community, such as employment, family/social relationships, legal issues, psychiatric and medical issues, and alcohol/drug use.

### Supplementary Information

**Additional file 1: **Supplemental Statistical Appendix.

## Data Availability

Statistical code: Available from Ms. Cadet (e-mail, tec4002@med.cornell.edu). Data set: Available in accordance with NIDA National Drug Abuse Treatment Clinical Trials Network policy (https://datashare.nida.nih.gov).

## Appendix

See Tables 3, 4, 5, 6

**Table 3:** Sensitivity Analyses

Characteristics	MI		Mean imputation		Case deletion		MICE	
	Odds ratio	P-value	Odds ratio	P-value	Odds ratio	P-value	Odds ratio	P-value
Mobility								
ASI Medical	0.718	**0.027**	0.884	0.123	0.848	0.245	0.857	0.208
ASI Legal	1.536	**0.028**	1.122	0.146	1.389	0.052	1.239	0.205
ASI Psychiatric	0.613	**0.022**	0.859	**0.035**	0.547	**0.027**	0.675	**0.047**
Follow-up Visit	0.547	0.393	0.279	**0.020**	0.107	0.254	0.103	0.135
Weeks abstinent	1.548	0.176	1.813	**0.000**	0.572	0.305	0.768	0.513
Randomization Group	0.194	**0.101**	0.694	0.364	0.047	**0.018**	0.097	**0.031**
Pain/Discomfort								
ASI Medical	0.683	**0.000**	0.781	**0.000**	0.767	**0.002**	0.770	**0.003**
ASI Psychiatric	0.741	**0.005**	0.830	**0.011**	0.696	**0.001**	0.690	**0.001**
Health Insurance	0.273	**0.031**	0.371	**0.002**	0.218	**0.047**	0.241	**0.036**
IDU history	2.683	0.082	2.155	0.067	3.604	0.048	3.094	0.053
Weeks Abstinent	1.151	0.548	1.333	**0.044**	0.922	0.826	1.043	0.891
Anxiety/Depression								
ASI Psychiatric	0.553	**0.000**	0.650	**0.000**	0.498	**0.000**	0.521	**0.000**
Weeks abstinent	1.384	0.146	1.533	**0.003**	1.073	0.800	1.014	0.957
Health Insurance	0.398	**0.049**	0.503	**0.047**	0.449	0.156	0.416	0.113
Randomization Group	2.858	**0.018**	2.224	0.011	1.582	0.338	1.895	0.174
HRQOL Utility Score								
	Coefficient	P-value	Coefficient	P-value	Coefficient	P-value	Coefficient	P-value
ASI Medical	− 0.016	**0.028**	− 0.008	0.103	− 0.015	0.025	− 0.013	0.064
ASI Family/Social Relationship	0.018	**0.014**	0.013	**0.009**	0.018	**0.005**	0.019	**0.006**
ASI Psychiatric	− 0.042	**0.000**	− 0.028	**0.000**	− 0.041	**0.000**	− 0.044	**0.000**
Weeks abstinent	0.068	**0.001**	0.027	**0.005**	− 0.008	0.592	0.021	0.321
Health Insurance	− 0.045	0.336	− 0.038	0.233	− 0.094	**0.020**	− 0.066	0.097
IDU history	0.065	0.210	0.039	0.312	0.097	**0.032**	0.071	0.100

Bolded values denotes statistical significance.

**Table 4:** Bivariate Analyses of Health Insurance Status

Characteristics	Health insurance (Yes = 1, No = 0)		
	Odds ratio	SE	P-value
Prescribed medication on regular basis (Yes = 1, No = 0)	0.49	0.14	0.01
Bothered by medical problems—past 30 days (Yes = 1, No = 0)	0.90	0.46	0.83
Admitted to hospital for any reason—since last assessment (Yes = 1, No = 0)	0.1	0.05	**0.000**
Visited medical office—since last assessment (Yes = 1, No = 0)	0.29	0.09	**0.000**
	Coefficient	SE	P-value
Days experienced medical problems—past 30 days	4.72	1.37	**0.000**

The first 4 bivariate analyses regressed on health insurance were analyzed using logistic regression. The number of days of experiencing medical problems was analyzed using OLS. Bolded values denotes statistical significance.

**Table 5:** Number of Missing Values in Imputed Variables

EQ-5D Characteristics	# of Missing values
Mobility	53/172
Anxiety/Depression	53/172
Pain/Discomfort	53/172
HRQoL Utility Score	53/172
ASI Characteristics	
Medical	56/172
Employment/support	55/172
Alcohol	56/172
Drug	55/172
Legal	55/172
Family/Social Support	55/172
Psychiatric	56/172

Table 5 displays the number of missing values for each imputed variable

**Table 6:** Analysis of Variance (ANOVA) for ASI Predictors

ASI Components	Coefficient	SE	P-value
Medical	0.114	0.111	0.355
Employment/Support	0.432	0.257	0.507
Alcohol	0.049	0.074	0.536
Drug	0.118	0.082	0.335
Legal	0.031	0.069	0.672
Family/Social Relationship	0.091	0.081	0.307
Psychiatric	0.153	0.104	0.247

ANOVA tests were conducted to evaluate if the imputed predictors variables (ASI scores) would differ significantly across separate imputation models
