# Supplementary material for: Determinants of health-related quality of life among individuals with opioid use disorder, recently released from incarceration
Source: Addict Sci Clin Pract. 2023 May 25;18:34. doi: 10.1186/s13722-023-00375-0 (PMC10210389; doi:10.1186/s13722-023-00375-0)
Supplement: Supplementary file 1 — Additional file 1: Supplemental Statistical Appendix. [file 13722_2023_375_MOESM1_ESM.docx]

**Supplemental Statistical Appendix**

**Missing Data Mechanism**

As a result of attrition (e.g., loss of contact, death, etc.), 64% (N=55) of 12-weeks post-release observations were missing, translating to approximately 32% of missing data in the overall sample. No missing data was observed at baseline. In order to evaluate the missing data mechanism (i.e., missing completely at random – MCAR; missing at random – MAR; missing not at random – MNAR), we first conducted Little’s MCAR test on the mobility, pain/discomfort, and anxiety/depression HRQoL variables.^49,52^ Results of the MCAR test indicated that the data were MCAR, both with and without the presence of auxiliary variables (p=1 and p= 0.65, respectively). However, because most of the missing observations were a consequence of participants missing their 12-week visit (i.e., unit missingness), 53/55 of the observations with missing data were automatically omitted from the MCAR test, potentially biasing the results towards a failure to reject the null hypothesis. Thus, to further assess whether the missing data were MAR vs. MCAR, logistic regression was conducted to evaluate whether other variables in the dataset could be used to determine the missingness of a particular variable.^52,70-72^ Results of the analyses indicated that several of the variables in the dataset could be used to predict missingness, thus supporting the MAR assumption. Although MAR vs. MNAR cannot be tested directly due to the nature of the missing data being unknown, we included auxiliary variables in our multiple imputation procedure to further support the MAR assumption, and conduct sensitivity analyses with regard to the method of analysis (see Table A1.)

**Imputation Models**

Imputed variables included all HRQoL outcome variables and ASI scores. Ordinal logistic regression was used in the imputation model for the HRQoL EQ-5D-5L domains because the domain response options contain ordered ranks.^73^ Four imputation models were run for each of the outcome variables, each containing all variables that would be included in the analytical model, as both variables to be imputed (i.e., ASI variables) and auxiliary variables.^47^ The number of iterations for the imputation model was set equivalent to the high fraction of missing information (FMI), based on imputation diagnostics.^70^ Once imputed, analysis was conducted on the pooled results of the imputed datasets.

One potential issue associated with imputing the models separately was that the imputed predictors variables (ASI scores) would differ significantly across models; thus, analysis of variance (ANOVA) tests were conducted to evaluate whether this was the case. Results of the ANOVA test revealed the values for the ASI predictor variables were not statistically different across the imputed datasets.

*Sensitivity Analyses*

As prior noted, the study had significant patient attrition. While MICE is the recommended approach to address jointly missing data in both dependent and independent variables,^47^ we explored how sensitive our results were to alternative imputation methods and complete case analysis. Mean imputation utilizing only the baseline ASI values as predictors and multiple imputations on the outcome variables only (HRQoL domains, HRQoL utility scores) were performed as additional sensitivity tests. Table A1 reports the comparative results across the three approaches. All of the statistically significant variables under MICE were also statistically significant across alternative imputation methods and case analysis, which supports the robustness assumption of our analytical model. Alternatively, the inverse was not observed, statistically significant variables under single variable imputation and mean imputation models were not statistically significant across other imputation models.
